# What Is Complex/Emotional About Emotional Complexity?

**DOI:** 10.3389/fpsyg.2019.01606

**Published:** 2019-07-12

**Authors:** Raul Berrios

**Affiliations:** Department of Management, Universidad de Santiago de Chile, Santiago, Chile

**Keywords:** emotional complexity, affect system, emotional features, complex systems, mixed emotions, meta-emotions, aesthetic emotions

## Abstract

Affective experiences can fluctuate, be combined, and fused, resulting in various phenomena labeled as being emotionally complex. Despite the lack of a common theoretical framework, several phenomena including mixed emotions, emodiversity, meta-emotions, awe, among several others, have been defined as being emotionally complex. In this conceptual analysis, I aim to integrate the diversity of emotional complexity by describing various phenomena associated with this construct. This integration offers a more comprehensive panorama of the current usage of the concept of emotional complexity compared to previous attempts to consolidate the field. Furthermore, this conceptual analysis intends to disentangle the emotional fingerprints of emotional complexity. In particular, I present evidence and arguments showing that complex emotions can be characterized as having specific facial expressions, appraisals, and functional significance. Finally, I suggest that it is possible to describe emotional complexity using concepts and properties from the complex systems theory. Concepts such as the hierarchical organization of the affect system and emergent self-organization are used to explain current evidence on emotional complexity. I explain that applying complex systems theory to emotional complexity is not only theoretically convenient, but that complex systems theory also serves to advance new forms to conceptualize the affect system. The current conceptual analysis can help to organize current research and theory in order to encourage new research endeavors in the field of emotional complexity and acknowledge the importance of emotional complexity in models of affect, for which I suggest some specific guidelines.

Emotions are sometimes more complex than the words we commonly use to express our feelings. Watching a loved one who is suffering acute pain pass away; being cheated while been a cheater; saying goodbye to friends when graduating. Life is full of occurrences when it is hard to communicate how we feel, but we can count these situations as wholly emotional.

In recent years, a burst but disperse number of research has emerged showing that our emotional life is complex (e.g., Lindquist and Barrett, 2010; Hay and Diehl, 2011; Grossman et al., 2016). Different phenomena including the co-activation of opposite emotions at the same time (e.g., mixed emotions) or the experience of feeling guilty for being happy observing others’ misfortune (i.e., meta-emotions) are forms of emotional complexity.

However, some obscurity remains in the literature when trying to comprehend a consensual definition of emotional complexity (Lindquist and Barrett, 2010; Grühn et al., 2013; Grossman et al., 2016). Furthermore, it is not clear what unifies different emotional experiences under the umbrella of emotional complexity. Likewise, no theoretical contribution in the field has accounted for the emotional or complex features of emotional complexity.

Hence, in this conceptual analysis, I address three issues related to emotional complexity and its role in emotional experience: (1) the diversity and apparent disparate number of phenomena characterized as emotional complexity, (2) the emotional fingerprints of emotional complexity, and (3) the complex nature of emotional complexity phenomena. I will present evidence and arguments showing that a unified emotional complexity framework is feasible, but it needs clarification of the emotional and complex features of emotional complexity.

## Revisiting the Concept of Emotional Complexity

Recently, Grossman et al. (2016) (see also Hay and Diehl, 2011) identified two different standard definitions of emotional complexity, which have resulted in disparate operationalization and measurement of the construct. They noticed that emotional complexity is generally understood either as emotional differentiation and emotional interdependence. These broad conceptualizations cover the majority of the common usages of the concept of emotional complexity in emotion science.

**Figure 1:**
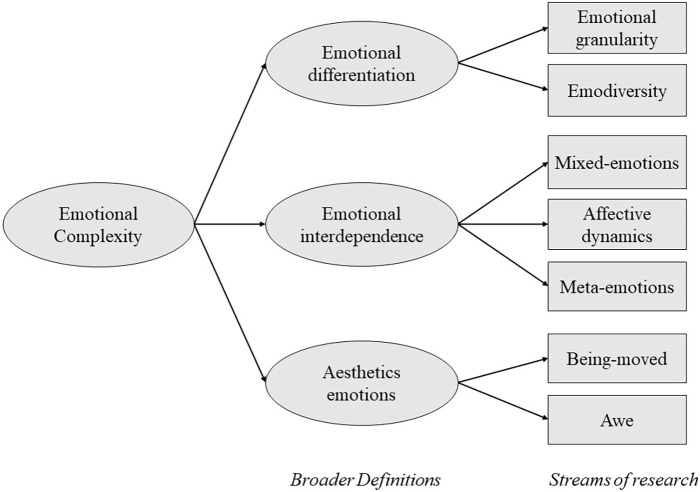
Representation of the common broad definitions of emotional complexity and some streams of research associated with them.

In this section, I expand on Grossman et al.’s distinction, identifying several streams of research that characterize the current usage of the concept of emotional complexity in the literature. The goal is not to provide a new definition of emotional complexity. Instead, I aim to succinctly describe some of the various phenomena related to emotional complexity, trying to identify a common theme across most of these phenomena. Figure 1 illustrates this diversity and organizes the broad definitions and some of the related streams of research.

### Emotional Complexity as Emotional Differentiation

One standard definition of emotional complexity concerns emotional differentiation. Emotional differentiation implies discerning among many positive and negative emotions (Grossman et al., 2016). In the emotional differentiation conceptualization of emotional complexity, it is possible to distinguish two different streams of research. One stream of research has considered subtle distinctions within emotional concepts as a meaningful expression of emotional complexity (Barrett, 2004; Kang and Shaver, 2004). For example, emotional granularity is an individual difference associated with the ability to make finer distinctions and well-differentiated reports of emotional experience, demonstrated by weak correlations between emotional states of the same valence (Barrett, 2004). Links between specific reports of emotional states and the corresponding subjective experience are substantiated in evidence showing that language contributes to the perception of emotions (Lindquist et al., 2006; Gendron et al., 2012).

The second stream of research conceptualizes emotional complexity as experiencing a broad and diverse range of different emotions (Quoidbach et al., 2014). The degree to which people can experience a diverse and abundant set of emotional experiences is a form of emotional complexity. Emodiversity is a measure of the richness of emotional complexity and the proportionality of experiences about a wide number of emotions (Quoidbach et al., 2014). Therefore, emodiversity and emotional granularity are at opposite sides of a hypothetical differentiation continuum, ranging from making thin distinctions between different emotions (emotional granularity) to experiencing an abundant and diverse range of emotions (emodiversity).

### Emotional Complexity as Emotional Interdependence

The other common definition understands emotional complexity as emotional interdependence (Grossman et al., 2016). Emotions mutually influence each other throughout an event, altering the intensity of subsequent affects, modifying the hedonic valence of ongoing experiences, coupling multiple emotional experiences as a consequence of similar appraisals, or changing the behaviors to be deployed at a given moment.

In the emotional interdependence conceptualization of emotional complexity, it is possible to distinguish three streams of research. First, interdependence can be conceptualized as the co-occurrence of emotional experiences of positive and negative valence (e.g., Larsen et al., 2001; Schimmack, 2001), such as in the experience of mixed emotions (e.g., happy-sad; fear-hope). Research has shown that students about to graduate experience more positive (happiness) and negative (sadness) emotions at the same time, compared to the same students surveyed at distant time points from graduation (Larsen et al., 2001; Hershfield et al., 2008).

Second, emotional interdependence is also defined as affective dynamics. Changes in emotion can result in diverse processes defined by the individuals’ fluctuations in emotion (Davidson, 1998). For example, emotional variability or instability is defined by the intraindividual variability of emotions over time (Grühn et al., 2013). Emotional variability involves marked fluctuation between different emotions, such as feeling excited and, not very long after, feeling blue. In this dynamic, emotions change dramatically from one moment to another.

These two streams of research are consistent with the main findings of Grühn et al. (2013). Their results indicate that there are four, mostly independent, factors of emotional complexity organized in nine time-based indicators of emotional complexity studied during seven consecutive days. Two factors resemble co-occurrence and affective dynamics. Co-occurrence is accounted for by the covariation between positive and negative emotions, whereas the overall variation in affective reports represents affective dynamics. The remaining two factors (positive and negative differentiation) reflect the conceptualization of emotional complexity as emotional differentiation, in particular, how discrete and precise are the emotional experiences reported by the people, ranging between highly differentiated (i.e., granularity), and abundant, richly componential (i.e., emodiversity).

The third stream of research conceptualizes emotional complexity as meta-emotions, with one emotion prompting a secondary emotion (Gottman et al., 1996; Norman and Furnes, 2016). I integrate meta-emotion within the definition of emotional complexity as interdependence because there is a teleological cause between a pair of affective experiences, which is a form of interdependence. Experiencing a meta-emotion requires that one emotion (e.g., sadness) triggers a secondary emotion (e.g., anger). Fundamental in the understanding of meta-emotions as a complex emotional experience is that emotions can be hierarchically organized, forming finite layers of emotions. Whereas in the previous conceptualizations, emotions can precede, follow, or coincide with another emotional experience, in meta-emotions, emotions are aggregated on top of each other.

These three streams of research (mixed emotions, affective dynamics, and meta-emotions) have a similar feature; that is, different emotions establish different interrelations between them. In mixed emotions, two oppositely valenced emotions co-occur, resulting in the subjective experience of two emotions as occurring at the same time (Berrios et al., 2015b). Emotion dynamics are characterized by multiple idiosyncratic patterns of reciprocal associations between different emotions, distinguishing the ebb and flow of the everyday emotional experience (Davidson, 1998). Finally, in meta-emotions, it is possible to observe a causal relation between a pair of different emotions; there is one emotion that serves as an object to trigger a secondary emotion (Norman and Furnes, 2016).

### Emotional Complexity as Aesthetic Emotions

Recent attempts to integrate the field of emotional complexity have not considered aesthetic emotions as a form of emotional complexity (e.g., Lindquist and Barrett, 2010; Grühn et al., 2013; Grossman et al., 2016). Aesthetic emotions are a group of affective experiences felt during aesthetic appreciation, including stimuli from nature (e.g., natural wonders) and human creation (e.g., painting or music), as well as emotional reactions that follow religious experiences or epiphanies (Keltner and Haidt, 2003; Gordon et al., 2016). These experiences are a form of emotional complexity, mostly because of the multiplicity of emotions involved and the difficulty to circumscribe them using single emotional words (Ortony et al., 1988; Keltner and Haidt, 2003).

Aesthetic emotions can be considered an independent category within this conceptualization of emotional complexity mostly because aesthetic emotions are a product of multiple emotions forming a synthesis. For example, research has shown that awe involves a mixture of surprise, pleasure, elevation, and astonishment (Keltner and Haidt, 2003).

Offering a complete characterization of the multiple streams of research on aesthetic emotions exceeds the goal of this section because many different emotional experiences have been studied. Thus, I introduce two complex emotional experiences commonly listed as aesthetic emotions: being-moved and awe.

Being-moved is a construct that is circumscribed to the arts and poetry, and only in recent years, it has been a subject of scientific exploration (Menninghaus et al., 2015). Menninghaus et al. (2015) have found that being-moved includes the emotional experiences of sadness and joy. Furthermore, they identified that critical life events, such as deaths and births, and significant relationship events (reunions) are the most common scenarios where this complex affective experience is triggered (Kuehnast et al., 2014). Finally, typical emotional appraisals observed when being-moved include high levels of compatibility with social norms and self-ideals. Frijda et al. (1989) also identified multiple cognitive appraisals when being-moved, including pleasantness, certainty, suddenness, importance, and other agency.

On the other hand, awe is a mixture of surprise, pleasure, elevation, and astonishment (Keltner and Haidt, 2003). Awe includes a feeling of wonder and amazement as a result of perceiving something vast that transcends our knowledge (Keltner and Haidt, 2003). In a recent study, Stellar et al. (2018) found that awe is preceded by appraisals of perception of vastness and need for accommodation (i.e., revise or create new mental schemas to account for paradoxical or unfamiliar information of the environment).

### A Common Theme Across the Different Streams of Research

From the three previously sketched conceptualizations of emotional complexity, versatility emerges as a distinct feature of emotional complexity. Versatility refers to the flexibility of the affect system when one is experiencing complex emotions. Versatility relates to the idea that, in emotional complexity, emotions are felt in multiple ways allowing individuals to integrate complex information, producing new verbalizations to communicate genuine feelings. Thus, for example, versatility is observed in emodiversity where experiencing a wide variety of emotions indicates greater elasticity of the affect system. Likewise, when people combine, aggregate, or fluctuate between different emotions, they reveal the vast flexibility of the human affect system, which allows them to “feel mixed” when graduating from school or “feel angry for being sad” after a romantic disappointment.

The idea that versatility is fundamental in understanding complex emotions is not new. For example, the Evaluative Space Model (ESM; Cacioppo et al., 1999, 2004) contends that positive affect and negative affect exist in distinct biological structures, which allow the independent activation of positive and negative emotions. An organism that processes both positive and negative emotions in parallel is capable of displaying a broader set of behaviors (i.e., versatility) appropriate to the circumstances.

This stance is also shared by the communicative model of emotion (Oatley and Johnson-Laird, 1996). Oatley and Johnson-Laird (1996) proposed that individuals react to events by making multiple cognitive evaluations, which in turn, may elicit complex emotions, giving rise to facial expressions that combine more than one basic emotion (i.e., versatility). Thus, versatility is as a property of the affect system in which cognitive and affective components of emotional experience (e.g., appraisals, valence) are flexibly integrated.

## What is Emotional in Emotional Complexity?

Missing from previous efforts to conceptualize emotional complexity (i.e., Lindquist and Barrett, 2010; Grossman et al., 2016) is a closer examination of the emotional fingerprints of emotional complexity. Indeed, what evidence do we have concerning the emotional signatures that accompany emotional complexity? Therefore, the aim in this section is to show indications that emotional complexity encompasses emotional signatures present in well-established definitions of emotion.

Defining the concept of emotion has been the subject of extensive debate in the history of Psychology (Gendron, 2010; Pérez-Almonacid, 2019). However, currently, it is mostly accepted that the concept of emotion is a description of its dominant uses, which implies a certain fuzziness and over inclusivity (Dixon, 2012). According to Mulligan and Scherer (2012), the minimum conditions that define an emotion are that: (1) emotions are directed toward an object; (2) emotions involve bodily changes that are felt; (3) emotions contain a subjective experience; (4) emotions are triggered by a particular evaluation of an external event, usually referred to as an appraisal; and (5) emotions have functional implications for individual and/or social life.

Emotions also produce consistent patterns of feelings over time that distinguish one individual from another (Gohm and Clore, 2000). Individual differences in emotions are states of feelings or moods that do not require an object (Clore et al., 1994). Emodiversity is an exemplar of individual differences in emotional complexity (Quoidbach et al., 2014).

### The Emotional Expression of Emotional Complexity

Previous research permits to describe three emotional fingerprints that characterize complex emotional experiences. First, during an emotionally complex episode, the affect system displays greater versatility, which produces characteristic facial expressions and physical reactions. Although facial expressions are not a definitive hallmark of the presence of a particular emotion (e.g., Russell, 1994), these are certainly an important marker of emotional experience in the literature (Keltner et al., 2003). Emotional expression can merge more than one gesture (Ekman and O’Sullivan, 1991; Kreibig et al., 2013, 2015; Du et al., 2014), and these expressions are combinations of emotions that have been thought to lie at opposite ends of the dimension of valence (c.f., Russell, 1980), such as disgust and joy.

New evidence supports previous incidental findings demonstrating that it is possible to identify 21 different and consistent facial expressions (Du et al., 2014), many of which reflect combinations of basic emotions (e.g., happily disgusted). Du et al. (2014) named all these combinations “compound emotions.” Pictures of 230 individuals’ emotional expression were taken during the elicitation of six basic emotions and 15 compound emotions, using imagery and images from previously validated studies. Through a computational model that automatically detects the shape of different features of the face, they showed that compound emotions are different from, but consistent with, the six basic emotions used in the study.

Likewise, recent research has shown that mixed emotions reflect specific facial muscle activation patterns that cannot be described merely as components of each emotion separately (Kreibig et al., 2013, 2015). Finally, some research on aesthetic emotions has shown that goose bumps or chills generally accompany awe and being-moved; these chills are not triggered by the componential emotions of being-moved, namely joy or sadness, but only by the complex experience of being-moved (Wassiliwizky et al., 2015).

### The Functional Significance and Appraisals of Emotional Complexity

It is also possible to suggest that complex emotions carry useful information that individuals use to appraise relevant events. According to some authors (Schwarz and Clore, 1983; Keltner and Haidt, 1999), emotions convey information that people use to interpret their current situation. Similarly, here, I propose that paradoxical events are typically those that elicit complex emotions. This assertion has found support in a number of recent studies showing that conflicting goals (e.g., wanting to finish your duties at the office, while at the same time wanting to get home earlier for a family dinner) typically yield the experience of mixed emotions (Berrios et al., 2015a, 2018a,b).

Menninghaus et al. (2015) also noted that events such as deaths, births, and reunions commonly elicit aesthetic emotions. Similarly, the experience of awe has been described as the need to accommodate new information either in the form of an active seeking of experiences that challenge current schemas (Shiota et al., 2006) or the level of uncertainty an individual experiences (Valdesolo and Graham, 2014). In all these cases, complex emotions are indicative of an event or information that is challenging, enigmatic, or disconcerting for one’s current mental schemas.

**Figure 2:**
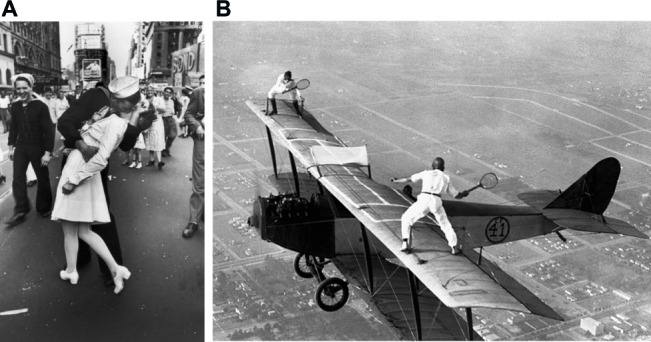
Graphical representation of the relationship between the degree of paradoxical information and the degree of versatility observed as complex emotions emerge. **(A)** “V-J Day in Times Square” (Eisenstaedt, 1945; w*ith permission of Getty Images*). This is an iconic photograph; no complex emotions are expected from its appreciation. **(B)** “Daredevils Playing Tennis on a Biplane” (Bettmann, 1925; w*ith permission of Getty Images*). Gladys Roy and Ivan Unger playing tennis on the wings of a biplane above Los Angeles.

Figure 2 shows a representation of the relationship between the degree of paradoxical information and the level of versatility manifested by the affect system when observing two different stimuli. The stimulus in the left down corner is very straightforward to understand; the degree of paradoxical information is almost zero. As a result, the affect system prompts fixed responses that lie within some form of positive affect. On the contrary, the stimulus in the upper right corner shows a paradoxical image. The photograph shows two men playing tennis on a biplane’s wings. The affect system triggers random responses, out of the standard spectrum that governs common emotional reactions (e.g., positive, negative), and as a result, one may feel anxious, surprised, and curious to determine whether the image is real (it is!).

Finally, it is possible to assert that appraisals when experiencing complex emotions are also complex. Appraisals mean that the evaluation of the surrounding circumstances of an affective experience plays an important role in the elicitation and differentiation of emotions (Arnold, 1960; Ellsworth and Scherer, 2003). Appraisals are perceptions of external events which are not related to high cognitive processing (Moors et al., 2013). As stated by Ellsworth and Scherer (2003), the “appraisals process is a link between the organism and the situation that produces the emotion” (p. 574). Thus, to fully consider the claim that paradoxical information is a meaningful driver of emotional complexity, it is necessary to account for particular appraisal processes when experiencing complex emotions.

Some evidence suggests that appraisals can be flexibly combined. For example, Smith and Ellsworth (1987) showed that when different appraisals are combined, it is possible to observe emotional blends (e.g., hope, challenge, and fear). They examined the appraisals and emotional reactions of individuals when taking an exam, and results revealed that combinations of patterns of appraisals are common during these stressful situations, following the elicitation of emotional blends. These preliminary data are consistent with the theory stating that the confluence of multiple emotions can control actions because each emotion contributes with multiple appraisals and motives regulating behavior (Frijda et al., 2014).

Additionally, Menninghaus et al. (2015) showed that the complex emotion of being-moved includes high ratings for appraisals of compatibility with social norms and self-ideals, showing that complex emotions may have distinct appraisals that assist individuals in the evaluation of the affective situation. Finally, Keltner and Haidt (2003) noticed that the prototypical cognitive appraisals associated with awe are the perception of vastness or self-diminishment and the need to mentally attempt to accommodate this vastness into existing mental schemas.

In sum, complex emotions have patterns of facial expression and physical reactions that are exclusively accounted for by the complexity of these emotional experiences, rather than the simple aggregation of affective components observed when experiencing a single emotion. Furthermore, complex emotions can include appraisals that characterize certain complex emotions, whereas other complex emotional experiences may involve the confluence of several different appraisals. The evidence revised so far also permits the assertion that complex emotions are functionally meaningful in signaling the presence of paradoxical information that challenges, puzzles, or disconcerts an individual’s current beliefs or mental schemas.

## What is Complex in Emotional Complexity?

There is an unexploited opportunity to apply the concepts and methods from complexity science to better examine the complex nature of emotional complexity. Complex systems theory can be defined as “an interdisciplinary field of research that seeks to explain how large numbers of relatively simple entities organize themselves, without the benefit of any central controller, into a collective whole that creates patterns, uses information, and, in some cases evolves and learns” (Mitchell, 2009: p. 4). Familiar exemplars of complex systems include the economies, and bee’s colonies.

According to Mitchell, a system is complex when “large networks of components with no central control and simple rules of operation give rise to complex collective behavior, sophisticated information processing, and adaptation via learning or evolution” (Mitchell, 2009: p. 13). Paralleling these ideas, we can call complex emotions insofar as the single components of the affect system (i.e., emotional adjectives) interact forming patterns or categories that are integrated into systems which do not resemble the constituent emotions permitting adaptive functions. As previously shown, specific properties of the affective experience, including emotional expression, functional significance, and appraisals reveal dynamics that exceed the rules observed when experiencing single emotions.

In this section, I intend to apply some of the most relevant properties of complex systems theory (Mitchell, 2009) to the study of emotional complexity. I explain some of these attributes regarding their relevance for current research and theory of emotional complexity. The goal is to refine the concept of emotional complexity in order to facilitate future research endeavors, choosing some properties of complex systems theory that best reflect the current evidence.

### Hierarchical Organization and the Emotional Lexicon

Spanish, English, or any other language, has a large number of terms that refer to emotions. Researchers usually simplify the structure of affect in order to explain the largest amount of variability using the smallest number of affective descriptors. These affective descriptors are used to account for the degree (or frequency) to which people experience a finite number of emotional adjectives. Language is essential in emotion research not only because it provides an essential research tool, but also because emotional words contribute to the subjective emotional experience itself (Barrett, 2004). In complex systems theory, languages and alphabets are considered forms of complex systems because multiple subsystems (e.g., words, codes) are tightly interrelated forming several new structures that actively communicate information (Simon, 1977).

**Figure 3:**
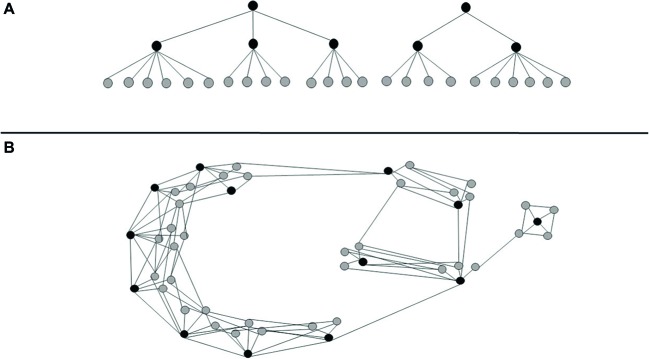
**(A)** Representation of common factorial models, resembling a tree structure, where the numerous emotional words are summed within increasingly parsimonious categories, and **(B)** new findings from Cowen and Keltner (2017) showing that the structure of affect is more complex, including several emotional categories and abundant interrelations across categories.

One common property of complex systems is the hierarchical organization. According to Simon (1977), a complex system is characterized by different levels, systems and subsystems distributed following the interrelation among the elements. Simon (1977) also specifies that interactions among near elements are stronger compared to elements at a more considerable distance. In the science of emotion, the most common characterization of affect is the tree structure, where closer elements reveal stronger associations (see Figure 3A). The tree-shaped structure is the observable organization that emerges from traditional factorial analysis. Although complex in appearance, this structure ignores different associations at other levels of interaction.

Shaver et al. (1987), in *Study 1*, investigated the hierarchical structure of affect using cluster analysis. They determined that the lowest level corresponds to the emotional lexicon that describes the language of a community of native speakers (213 emotional adjectives surveyed). In the next upper level, two smaller sets of discrete emotions were described (love, joy, surprise, anger, sadness, and fear). These more or less correspond to the basic emotional adjectives found by theorists of basic emotions.

The hierarchical structure described by Shaver et al. (1987) also distinguishes two broad characterizations of emotions as positive affect and negative affect at the top of the structure. Popular theories of affect, such as the circumplex model of affect (Russell, 1980), state that positive affect and negative affect represent opposite ends of a bipolar dimension of valence.

However, the emotional lexicon, when referring to most of the emotional complexity phenomena, seems to be more intricate. There are not many words to account for experiences such as mixed emotions, meta-emotions, or awe. Characterizations of affective life, either using basic emotions or overall dimensions of positive affect and negative affect, do not resemble the affect system when experiencing complex emotions. For example, Table 1 shows different verbalizations of some complex emotional experiences in three languages (English, Spanish, and Portuguese). It is clear from the examples that these are genuine affective experience, but only some of them can be described using regular emotional words (e.g., happy, sad).

**Table 1:** Exemplars of common emotionally complex, linguistic expressions in English, Spanish, and Portuguese.

	English	Spanish	Portuguese
Co-activation of emotions	Mixed feelings	sentimientos encontrados	sentimentos misturados
	Tears of joy	lágrimas de alegría	lágrimas de felicidade
	Being-moved	conmovido	movido
Meta-emotion	Feeling guilty for feeling good	Sentirse culpable por alegrarse de la desgracia ajena	Sentindo-se culpado pela nossa alegria maliciosa
	I hate how much I love you	Odio cuanto te amo	Eu odeio o quanto eu te amo
	The joy of being sad	La alegría de estar triste	A alegria de estar triste

Simon (1977) contends that the observable associations in a system ignore the detailed structure at other levels of interaction. He exemplified this idea by explaining that the middle band of frequencies only determines the observable dynamics of the system (i.e., sounds we can hear). The structure of interactions in other subsystems is nearly independent or “nearly decomposable” at the next level (i.e., high or low frequencies; Simon, 1977). The affect system can be then also nearly decomposable. Emotional expressions such as mixed feelings or tears of joys are clearly out of the spectrum, but they are still genuine feelings, with identifiable emotional components.

### Structures and (Non-)additive Models of Affect

Conventional representations of the affect system are different versions of factorial models, in which the abundant emotional lexicon is simplified to obtain more parsimonious descriptors of the emotional experience. These models can be categorized as *additive models of affect*. That is to say that, for example, emotional adjectives including happiness, joy, and excitement are grouped under the common name of positive emotions. On the other hand, emotional adjectives such as sadness, sorrow, and blue are classified as negative emotions. Following dimensional models of affect, all positive emotions and all negative emotions share the same affective valence (positive and negative, correspondingly).

The structure of affect following additive principles is then limited to a small number of varieties of emotional experience. However, in order to account for complex emotional experiences, it is necessary that multiple interrelations across categories could represent emotional lexicon. In other words, to describe complex emotions, it is necessary to observe both a large number of distinct categories of emotional experience and strong networks across some of these categories. Thus, the classic factorial, tree structure needs to be subverted, giving place to new forms to represent the emotional experience.

Precisely, Cowen and Keltner (2017) investigated the taxonomy of emotional experience. In their study, 853 participants viewed a subsample of 30 film clips designed to elicit a wide variety of feelings (e.g., awe, disgust, melancholy). They also innovated in the mathematical framework using canonical correlation analysis in order to characterize the emotional experience as points within a semantic space, distributed along dimensions.

Their results showed that the semantic space is far more abundant than previously thought. Cowen and Keltner (2017) found 27 distinct varieties of emotional experience. This evidence elegantly coincides with the 21 different and consistent facial expressions found by Du et al. (2014). Evidence also showed that there are abundant interrelations between the 27 varieties of emotional experience, revealing continuous gradients between categories, rather than discrete, independent emotions or rigid dimensions of emotions. A simplified representation of the graphic map produced by Cowen and Keltner (2017) can be seen in Figure 3B.

These results suggest that it is reasonable to think that complex emotions are the result of new forms of organization of the affect system. For some *additive models of affect*, connections across categories are interpreted as measurement error, but here, I propose that connections across categories can construe subjectively meaningful emotional experiences, which we can accurately call complex emotions. In fact, recent research adds to this claim showing that emotional categories identified by subjects from five different cultures based on more than 2,000 speech samples can communicate at least 12 different categories of emotions, forming a structure connected by different blends of emotions (Cowen et al., 2019), which do not resemble the tree-shaped structure of the affect system.

Cowen and Keltner’s findings concerning the observation of fuzzy boundaries across categories also suggest that complex emotions can be forms of *non-additive models of affect*, where the experience of certain complex emotions is a function of different relations among multiple categories of affective experience. Thus, for example, the experience of awe is not merely the result of the linear addition of surprise, astonishment, elevation, and pleasure, but a form of a more complex dynamic which results in an affective experience that is subjectively and physically distinct.

### Emotional Complexity as an Emergent Self-Organization Phenomenon

Heylighen (1989) argues that emergence refers to properties of higher order structures that cannot be reduced to their constituents parts. Heylighen (1989) also adds that two critical characteristics of emergent dynamics are self-organization and the hierarchical or multilevel structure of the systems. Notions of hierarchical organization in complex systems and how this applies to emotional complexity have been already explained.

Self-organization, on the other hand, is understood as a spontaneous process of organization (Heylighen, 1989). Spontaneous means that multiple control systems reduce a state of chaos (i.e., disorganization) *via* continuous negative feedback loops among multiple systems (Guastello, 2002). An example of this is a flock of birds moving over the sky – they can form a qualitatively stable structure (i.e., shape) by multiple feedback loops among the individual animals; from an apparent state of chaos emerges a qualitatively stable structure that does not require cognizant awareness of the relationships between individuals and the collective.

Introducing the idea that emotional complexity can be understood as an emergent self-organization phenomenon departs from the fact that when experiencing complex emotions, the emotional lexicon is increasingly integrated, facilitating the emergence of *sui generis* emotional expressions. Thus, experiencing complex emotions may result in new verbalizations of emotions, uncommon in the emotional lexicon (e.g., mixed feelings), or the combination of multiple emotional adjectives into one single experience such as *awe*. Like in the case of the flock of birds, in emotional complexity, emotional words no longer reflect the current state of feelings, but they are mixed, combined, or intertwined in such a way that a new structure emerges, with identifiable emotional properties that are more than the single emotional words.

Emodiversity (Quoidbach et al., 2014) is an excellent example of complex emotions that follows principles of self-organization. Quidbach and colleagues sampled more than 300,000 observations and observed that people vary in terms of the number of emotional experiences they report on a given day. They found that the variety and relative abundance of the emotions people experience is an integral component that distinguishes the emotional experience between individuals. Emodiversity was also found to predict better physical and mental health. Quoidbach et al. (2014) used Shannon entropy equation to quantify the degree of emodiversity for each participant in the study. Shannon entropy quantifies the probability distribution of a set of elements (i.e., emotions; Ladyman et al., 2013). Therefore, emodiversity is a quantification of the chaos in a system, and the subsequent stability that can emerge as a result of the probability distribution of the components.

Other studies also suggest that complex emotions may be emergent self-organization phenomena. For example, Berrios et al. (2018a) showed that indices of mixed emotions are positively correlated both with positive and negative single emotions. These data reveal a discontinuity from the typical negative correlations observed between positive and negative emotions. Similarly, Kreibig et al. (2013, 2015) demonstrated that the experience of mixed emotions is generally accompanied by specific patterns of activation of facial muscles that combined oppositely valenced emotions (e.g., amused and disgust), and these patterns are different from those observed in single emotion expressions. Again, the whole is greater than the sum of the parts, suggesting that it is possible to interpret complex emotions as emergent self-organization phenomena.

## Conclusions

Research and theory of emotional complexity have produced relevant data and innovative propositions that challenge traditional conceptualizations of the affect system. However, important tenets of the concept of emotional complexity itself have not been sufficiently addressed.

Previous efforts to theorize on emotional complexity have tried to bond a standard definition (Lindquist and Barrett, 2010), but they have not considered several recent phenomena studied (e.g., meta-emotions, emodiversity). Other scholars have tried to synthesize the diversity of definitions creating a conceptualization that takes into account specific features of emotional complexity (e.g., interrelations between emotions, differentiation, temporal dynamics of emotions; Grühn et al., 2013; Grossman et al., 2016). Still, there are phenomena such as meta-emotions and aesthetic emotions that have not been integrated into a unified framework, and no previous conceptualization of emotional complexity has explored the emotional and complex features of emotional complexity.

As a result, the goal of the present conceptual analysis was twofold. Firstly, I have tried to integrate the diversity of emotional complexity by describing the various phenomena associated with this construct. Secondly, I have disentangled the specific emotional components and complex features of emotional complexity. This conceptual analysis can, then, further a research agenda and facilitate future research initiatives and current studies using emotional constructs in situations where it is feasible to observe complex emotions.

Relevant available research was used to justify two main propositions. First, I sustain that emotional complexity entails some specific emotional signatures that are common in well-established definitions of emotion. Emotional complexity includes particular facial and physical reactions. These facial and physical reactions are better characterized by the complexity of the emotional experience, rather than by the single emotional constituents of a complex emotion. Furthermore, several studies point to the idea that emotional complexity is functional to situations involving paradoxical or puzzling information that individuals need to process. This claim anchors in the observation that several complex emotions include the confluence of multiple appraisals, which is also accounted for by the complex experience itself.

Second, so far, research and theory on emotional complexity have not sufficiently explained the complex nature of complex emotions. In order to commence to remedy this situation, I suggest that it is possible to describe emotional complexity using concepts and methods from the complex systems theory. Specifically, emotional words are essential elements that can form different structures and organization systems. The simplest, observable subsystem is the traditional tree-shaped structure obtained using factorial analysis. This structure determines specific properties (e.g., valence) that accurately describe several of everyday emotional experiences.

However, as suggested by Simon (1977), other structures can be described, which not necessarily resemble the observable system. Thus, the same single emotional adjectives can be related in multiple forms, producing other subsystems. Recent evidence supports this idea showing that the emotional lexicon can produce 27 categories based on multiple interrelations (Cowen and Keltner, 2017) and that the structure of affect is connected by various emotional blends between categories (Cowen et al., 2019).

The organization of the affect system in the form suggested by Cowen and Keltner (2017) permits to sustain that several complex emotional experiences are feasible to be located in this new representation of affective life. Mixed emotions (i.e., the co-activation of a pair of oppositely valenced emotions); meta-emotions (i.e., the activation of two emotions, where one emotion is used as an object to experiencing a secondary emotion); and awe (i.e., the co-activation of more than one emotion, generally opposite in valence) are genuine feelings that subvert the common tree-shaped representation of affect. In all these examples (as well as in most of the phenomena commented in the present conceptual analysis), the complex emotional experience, as a whole, is greater than the constituents, single emotions.

### Suggestions for Future Research on Emotional Complexity

Although the general consensus between specialists acknowledges the existence of emotional complexity as an overarching concept (e.g., Lindquist and Barrett, 2010), research is still scattered and not neatly integrated. One of the challenges for researchers interested in emotional complexity is to adhere to some basic conceptual tenets, susceptible to be tested in future research. The general expectation of this conceptual analysis is to contribute to the efforts to consolidate a unified field of study on emotional complexity, which can reinvigorate the interest of researchers in the intricacy of our emotional life.

In this regard, three suggestions can be offered. First, it is necessary to determine the differences or equivalence between complex emotions. For example, it has been suggested that mixed emotions may be a form of meta-emotions (Russell, 2017). Although these concepts are conceptually and experientially different (i.e., mixed emotions involve the co-activation of two oppositely valenced emotions, whereas meta-emotions involve an emotion used as a trigger of a secondary emotion), distinguishing between these phenomena may provide new tools to apply complex systems theory in the study of emotional complexity.

Future research should better justify the applicability of the emotional complexity phenomena in the context of specific research. For example, regarding the differentiation of mixed emotions and meta-emotions, it is vital to justify under which circumstance people are more probable to experience mixed emotions or meta-emotions (if they are different). Mixed emotions have mostly been observed in the context of goal conflict (Berrios et al., 2015a, 2018b), whereas meta-emotional experiences are more likely when people are paying more considerable attention to emotion (Bailen et al., 2018). These previous studies may suggest that mixed emotions are more instrumental experiences that respond to conflicting demands or expectations, whereas meta-emotions are more self-monitoring experiences that result from appraising our behavior. This type of distinction is still awaiting further research, but generally, researchers should devote more efforts when justifying the pertinence of the chosen complex emotional phenomenon.

The second suggestion derived from this conceptual analysis is a call for researchers to test for complex emotions when conducting studies involving situations or manipulations where it is feasible to observe some form of emotional complexity (i.e., situations or manipulations with increasing levels of paradoxical information). Although perhaps required in the near future, it is not currently necessary to implement sophisticated calculations to evaluate the influence of complex emotions on some outcomes. For example, when a researcher uses emotional adjectives to measure an affect-related construct, it would be feasible to examine the impact of some complex emotions, such as mixed emotions, if the situation or experimental manipulation involves conflict between goals (e.g., Berrios et al., 2015a).

Furthermore, introducing complex emotions as covariates is a highly recommended practice in future studies. The simplest form to include complex emotions is by testing the interaction between positive affect and negative affect in the model with covariates. If a significant effect is found or it turns out that the interaction acts as a confounder, this may suggest that a more complex feature of emotional experience is in place, which can stimulate the curiosity of researchers interested in the complexity of the emotional experience. Another form to test for complex emotions is to compute simple indices of mixed emotions, such as the minimum value (Schimmack, 2001). Finally, it could be possible to test for individual differences in emodiversity in the model, which can be implemented following the guidelines and code provided by Quoidbach et al. (2014).

Finally, in order to advance models based on the complex systems theory (Simon, 1977; Ladyman et al., 2013), new methods and techniques are needed. Important steps toward this direction have been taken by Cowen and colleagues (Cowen and Keltner, 2017; Cowen et al., 2019), who by implementing new mathematical techniques have shown new forms to represent our affective life, beyond the tree-shaped structure.

One alternative might be to describe interrelations among emotional adjectives as different forms of simplices. In algebraic topology, an *n*-simplex is a generalization of a geometric space to *n* dimensions (May, 1999). Simplices describe the structure of a group of elements, such that a simplex *n* = 0 is a single node, *n* = 1 is a straight line, and *n* = 2 is a triangle. Thus, for example, combinations of *n*-simplices without recursive paths might describe the structure of meta-emotions, where the boundary of the paths is the difference between each pair of emotional adjectives. Here, one emotion must follow another one in an orderly sequence, similar to the experience of meta-emotions. Mixed emotions, on the other hand, could be represented as cycles, such that when the boundary of the paths of *n*-simplices equals zero, the structure is a cycle, involving recursive paths between emotional adjectives. The recursive paths reflect the interdependence of emotional adjectives of opposite valence, without a given order.

Similar propositions have been advanced by Heylighen (1989). He noted that the study of emergent self-organized systems could be represented using algebraic transformations of groups of relations, labeled as types of closures. Heylighen (1989) proposed four types of closure: recursive, cyclical, surjective (many-to-one), and inverse surjective (one-to-many). Moreover, the combination of them may result in several network topologies.

Overall, the application of complex systems theory to the study of emotional complexity is both intuitive and challenging. Emotional features identified in this conceptual analysis suggest that it is possible to distinguish complex emotions as experiences where the whole is greater than the sum of its parts. However, implementing new approaches and methods to explore the underlying structure of the affect system still need further research (although Cowen and Keltner, 2017). In any case, future research in emotional complexity may need to substitute classic models of affect, and start to explore the benefits of complexity when studying the emotional life. It is necessary to raise attention of researchers investigating emotional processes about the potential benefits of studying emotional complexity for gaining richer information of our current models of affect. Emotional complexity should not be considered as random variance without a fair examination guided by informed research and theory.

## Author Contributions

The author confirms being the sole contributor of this work and has approved it for publication.

### Conflict of Interest Statement

The author declares that the research was conducted in the absence of any commercial or financial relationships that could be construed as a potential conflict of interest.

## References

[ref1] Arnold M. B. (1960). Emotion and personality. New York, NY, US: Columbia University Press.

[ref2] Bailen N. H. Wu H. Thompson R. J. (2018). Meta-emotions in daily life: associations with emotional awareness and depression. Emotion 10.1037/emo0000488 Advance online publication. 30080076

[ref3] Barrett L. F. (2004). Feelings or words? Understanding the content in self-report ratings of experienced emotion. J. Pers. Soc. Psychol. 87, 266–281. 10.1037/0022-3514.87.2.266, PMID: 15301632 PMC1351136

[ref4] Berrios R. Totterdell A. Kellett S. (2015a). Investigating goal conflict as a source of mixed emotions. Cognit. Emot. 29, 755–763. 10.1080/02699931.2014.939948 25040183

[ref5] Berrios R. Totterdell A. Kellett S. (2015b). Eliciting mixed emotions: a meta-analysis comparing models, types, and measures. Front. Psychol. 6, 1–15. 10.3389/fpsyg.2015.00428 25926805 PMC4397957

[ref6] Berrios R. Totterdell P. Kellett S. (2018a). When feeling mixed can be meaningful: the relation between mixed emotions and eudaimonic well-being. J. Happiness Stud. 19, 841–861. 10.1007/s10902-017-9849-y

[ref7] Berrios R. Totterdell P. Kellett S. (2018b). Silver linings in the face of temptations: how mixed emotions promote self-control efforts in response to goal conflict. Motiv. Emot. 42, 909–919. 10.1007/s11031-018-9707-1

[ref8] Bettmann (Daredevils Playing Tennis on a Biplane). (1925). Getty Images. *Editorial rights under order number 2059468448 to Raul Berrios*.

[ref9] Cacioppo J. T. Gardner W. L. Berntson G. G. (1999). The affect system has parallel and integrative processing components: form follows function. J. Pers. Soc. Psychol. 76, 839–855. 10.1037/0022-3514.76.5.839

[ref10] Cacioppo J. T. Larsen J. T. Smith N. K. Berntson G. G. (2004). “The affect system: what lurks below the surface of feelings?” in Feelings and emotions: The Amsterdam symposium. eds. Manstead A. S. R. Frijida N. Fisher A. (New York, NY: Cambridge University Press), 223–242.

[ref11] Clore G. L. Schwarz N. Conway M. (1994). “Affective causes and consequences of social information processing” in Handbook of social cognition. 2nd Edn. eds. Wyer R. S. Srull T. K., vol. 1 (Hillsdale, NJ: Lawrence Erlbaum), 323–418.

[ref12] Cowen A. S. Keltner D. (2017). Self-report captures 27 distinct categories of emotion bridged by continuous gradients. Proc. Natl. Acad. Sci. USA 114, E7900–E7909. 10.1073/pnas.1702247114, PMID: 28874542 PMC5617253

[ref13] Cowen A. S. Laukka P. Elfenbein H. A. Liu R. Keltner D. (2019). The primacy of categories in the recognition of 12 emotions in speech prosody across two cultures. Nat. Hum. Behav. 3, 369–3821. 10.1038/s41562-019-0533-6, PMID: 30971794 PMC6687085

[ref14] Davidson R. J. (1998). Affective styles and affective disorders: perspectives from affective neuroscience. Cognit. Emot. 12, 307–330. 10.1080/026999398379628

[ref15] Dixon T. (2012). “Emotion”: the history of a keyword in crisis. Emot. Rev. 4, 338–344. 10.1177/1754073912445814 23459790 PMC3573683

[ref16] Du S. Tao Y. Martinez A. M. (2014). Compound facial expressions of emotion. Proc. Natl. Acad. Sci. USA 111, E1454–E1462. 10.1073/pnas.1322355111, PMID: 24706770 PMC3992629

[ref17] Eisenstaedt A. (V-J Day in Times Square). (1945). Time & Life Pictures/Getty Images. *Editorial rights under order number 2056291862 to Raul Berrios*. PMC5218264 29015821

[ref18] Ekman P. O’Sullivan M. (1991). Who can catch a liar? Am. Psychol. 46, 913–920. 1958011 10.1037//0003-066x.46.9.913

[ref19] Ellsworth P. C. Scherer K. R. (2003). “Appraisal processes in emotion” in Handbook of affective sciences. eds. Davidson R. J. Scherer K. R. Hill Goldsmith H. (New York, NY: Oxford University Press), 572–595.

[ref20] Frijda N. Kuipers P. Ter Schure E. (1989). Relations among emotion, appraisal, and emotional action readiness. J. Pers. Soc. Psychol. 57, 212–228. 10.1037//0022-3514.57.2.212

[ref21] Frijda N. H. Ridderinkhof K. R. Rietveld E. (2014). Impulsive action: emotional impulses and their control. Front. Psychol. 5, 1–9. 10.3389/fpsyg.2014.00518 24917835 PMC4040919

[ref22] Gendron M. (2010). Defining emotion: a brief history. Emot. Rev. 2, 371–372. 10.1177/1754073910374669

[ref23] Gendron M. Lindquist K. A. Barsalou L. Barrett L. F. (2012). Emotion words shape emotion percepts. Emotion 12, 314–325. 10.1037/a0026007 22309717 PMC4445832

[ref24] Gohm C. L. Clore G. L. (2000). Individual differences in emotional experience: mapping available scales to processes. Personal. Soc. Psychol. Bull. 26, 679–697. 10.1177/0146167200268004

[ref25] Gordon A. M. Stellar J. E. Anderson C. L. McNeil G. D. Loew D. Keltner D. (2016). The dark side of the sublime: distinguishing a threat-based variant of awe. J. Pers. Soc. Psychol. 113, 310–328. 10.1037/pspp0000120 27929301

[ref900] Gottman J. M. Katz L. F. Hooven C. (1996). Parental meta-emotion philosophy and the emotional life of families: Theoretical models and preliminary data. J. Fam. Psychol. 10, 243–268.

[ref26] Grossman I. Huynh A. C. Ellsworth P. C. (2016). Emotional complexity: clarifying definitions and cultural correlates. J. Pers. Soc. Psychol. 111, 895–916. 10.1037/pspp0000084, PMID: 26692354

[ref27] Grühn D. Lumley M. A. Diehl M. Labouvie-Vief G. (2013). Time-based indicators of emotional complexity: interrelations and correlates. Emotion 13, 226–237. 10.1037/a0030363, PMID: 23163712 PMC3600390

[ref28] Guastello S. J. (2002). Managing emergent phenomena: Nonlinear dynamics in work organizations. New York, NY, US: Psychology Press.

[ref29] Hay E. L. Diehl M. (2011). Emotion complexity and emotion regulation across adulthood. Eur. J. Ageing 8, 157–168. 10.1007/s10433-011-0191-7, PMID: 21941465 PMC3176462

[ref30] Hershfield H. Mikels J. A. Sullivan S. J. Carstensen L. L. (2008). Poignancy: Mixed emotional experience in the face of meaningful ending. J. Pers. Soc. Psychol. 94, 158–167. 10.1037/0022-3514.94.1.158, PMID: 18179325 PMC2807633

[ref31] Heylighen F. (1989). “Self-organization, emergence and the architecture of complexity” in Proceedings of the 1st European conference on system science. Vol. 18, (Paris: AFCET), 23–32.

[ref32] Kang S. M. Shaver P. R. (2004). Individual differences in emotional complexity: their psychological implications. J. Pers. 72, 687–726. 10.1111/j.0022-3506.2004.00277.x 15210014

[ref33] Keltner D. Ekman P. Gonzaga G. Beer J. (2003). “Facial expression of emotion” in Handbook of affective sciences. eds. Davidson R. J. Scherer K. R. Goldsmith H. H. (New York, NY, US: Oxford University Press), 415–432.

[ref34] Keltner D. Haidt J. (1999). Social functions of emotions at multiple levels of analysis. Cognit. Emot. 13, 505–522. 10.1080/026999399379168

[ref35] Keltner D. Haidt J. (2003). Approaching awe, a moral, spiritual, and aesthetic emotion. Cognit. Emot. 17, 297–314. 10.1080/02699930302297 29715721

[ref36] Kreibig S. D. Samson A. C. Gross J. J. (2013). The psychophysiology of mixed emotional states. Psychophysiology 50, 799–811. 10.1111/psyp.12064, PMID: 23730872

[ref37] Kreibig S. D. Samson A. C. Gross J. J. (2015). The psychophysiology of mixed emotional states: Internal and external replicability analysis of a direct replication study. Psychophysiology 52, 873–886. 10.1111/psyp.12425, PMID: 25959633

[ref38] Kuehnast M. Wagner V. Wassiliwizky E. Jacobsen T. Menninghaus W. (2014). Being moved: linguistic representation and conceptual structure. Front. Psychol. 5, 1–11. 10.3389/fpsyg.2014.01242 25404924 PMC4217337

[ref39] Ladyman J. Lambert J. Wiesner K. (2013). What is a complex system? Eur. J. Philos. Sci. 3, 33–67. 10.1007/s13194-012-0056-8

[ref40] Larsen J. T. McGraw A. P. Cacioppo J. T. (2001). Can people feel happy and sad at the same time? J. Pers. Soc. Psychol. 81, 684–696. 10.1037/00223514.81.4.684 11642354

[ref41] Lindquist K. A. Barrett L. F. (2010). “Emotional complexity” in The handbook of emotions. eds. Lewis M. Haviland-Jones J. M. Barrett L. F. (New York, NY: Guilford Press), 513–530.

[ref42] Lindquist K. A. Barrett L. F. Bliss-Moreau E. Russell J. A. (2006). Language and the perception of emotion. Emotion 6, 125–138. 10.1037/1528-3542.6.1.125, PMID: 16637756

[ref43] May J. P. (1999). A concise course on algebraic topology. Chicago, IL: University of Chicago Press.

[ref44] Menninghaus W. Wagner V. Hanich J. Wassiliwizky E. Kuehnast M. Jacobsen T. (2015). Towards a psychological construct of being moved. PLoS One 10, 1–33. 10.1371/journal.pone.0128451 PMC4456364 26042816

[ref45] Mitchell M. (2009). Complexity: A guided tour. New York: Oxford University Press.

[ref46] Moors A. Ellsworth P. C. Scherer K. R. Frijda N. H. (2013). Appraisal theories of emotion: state of the art and future development. Emot. Rev. 5, 119–124. 10.1177/1754073912468165

[ref47] Mulligan K. Scherer K. R. (2012). Toward a working definition of emotion. Emot. Rev. 4, 345–357. 10.1177/1754073912445818

[ref48] Norman E. Furnes B. (2016). The concept of “metaemotion”: what is there to learn from research on metacognition? Emot. Rev. 8, 187–193. 10.1177/1754073914552913 27110281 PMC4820014

[ref49] Oatley K. Johnson-Laird P. N. (1996). “The communicative theory of emotions: empirical tests, mental models, and implications for social interaction” in Striving and feeling. Interaction among goals, affect, and self-regulation. eds. Martin L. Tesser A. (New York, NY: Russell Sage), 363–393.

[ref50] Ortony A. Clore G. L. Collins A. (1988). The cognitive structure of emotions. New York: Cambridge University Press.

[ref51] Pérez-Almonacid R. (2019). A non-mediational approach to emotions and feelings. Front. Psychol. 10:181. 10.3389/fpsyg.2019.00181 30804848 PMC6370897

[ref52] Quoidbach J. Gruber J. Mikolajczak M. Kogan A. Kotsou I. Norton M. I. (2014). Emodiversity and the emotional ecosystem. J. Exp. Psychol. Gen. 143, 2057–2066. 10.1037/a0038025 25285428

[ref53] Russell J. A. (1980). A circumplex model of affect. J. Pers. Soc. Psychol. 39, 1161–1178. 10.1037/h0077714

[ref54] Russell J. A. (1994). Is there universal recognition of emotion from facial expression? A review of the cross-cultural studies. Psychol. Bull. 115, 102–141. 10.1037/0033-2909.115.1.102, PMID: 8202574

[ref55] Russell J. A. (2017). Mixed emotions viewed from the psychological constructionist perspective. Emot. Rev. 9, 111–117. 10.1177/1754073916639658

[ref56] Schimmack U. (2001). Pleasure, displeasure, and mixed feelings: are semantic opposites mutually exclusive? Cognit. Emot. 15, 81–97. 10.1080/02699930126097

[ref57] Schwarz N. Clore G. L. (1983). Mood, misattribution, and judgments of well-being: informative and directive functions of affective states. J. Pers. Soc. Psychol. 45, 513–523. 10.1037/0022-3514.45.3.513

[ref58] Shaver P. Schwartz J. Kirson D. O’Connor C. (1987). Emotion knowledge: further exploration of a prototype approach. J. Pers. Soc. Psychol. 52, 1061–1086. 10.1037/0022-3514.52.6.1061 3598857

[ref59] Shiota M. N. Keltner D. John O. P. (2006). Positive emotion dispositions differentially associated with big five personality and attachment style. J. Posit. Psychol. 1, 61–71. 10.1080/17439760500510833

[ref60] Simon H. A. (1977). “The organization of complex systems” in Models of discovery. Boston studies in the philosophy of science. Vol 54. (Dordrecht: Springer), 245–261.

[ref61] Smith C. A. Ellsworth P. C. (1987). Patterns of appraisal and emotion related to taking an exam. J. Pers. Soc. Psychol. 52, 475–488. 10.1037/0022-3514.52.3.475, PMID: 3572720

[ref62] Stellar J. E. Gordon A. Anderson C. L. Piff P. K. McNeil G. D. Keltner D. (2018). Awe and humility. J. Pers. Soc. Psychol. 114, 258–269. 10.1037/pspi0000109, PMID: 28857578

[ref63] Valdesolo P. Graham J. (2014). Awe, uncertainty, and agency detection. Psychol. Sci. 25, 170–178. 10.1177/0956797613501884 24247728

[ref64] Wassiliwizky E. Wagner V. Jacobsen T. Menninghaus W. (2015). Art-elicited chills indicate states of being moved. Psychol. Aesthet. Creat. Arts 9, 405–416. 10.1037/aca0000023

